# HIV/Mtb Co-Infection: From the Amplification of Disease Pathogenesis to an “Emerging Syndemic”

**DOI:** 10.3390/microorganisms11040853

**Published:** 2023-03-27

**Authors:** José Miguel Azevedo-Pereira, David Pires, Marta Calado, Manoj Mandal, Quirina Santos-Costa, Elsa Anes

**Affiliations:** 1 Host-Pathogen Interactions Unit, Research Institute for Medicines, iMed-ULisboa, Faculty of Pharmacy, Universidade de Lisboa, Av. Prof. Gama Pinto, 1649-003 Lisboa, Portugal; 2 Center for Interdisciplinary Research in Health, Católica Medical School, Universidade Católica Portuguesa, Estrada Octávio Pato, 2635-631 Rio de Mouro, Portugal

**Keywords:** tuberculosis, HIV, granuloma, immunodeficiency, co-infection

## Abstract

Human immunodeficiency virus (HIV) and *Mycobacterium tuberculosis* (Mtb) are pathogens responsible for millions of new infections each year; together, they cause high morbidity and mortality worldwide. In addition, late-stage HIV infection increases the risk of developing tuberculosis (TB) by a factor of 20 in latently infected people, and even patients with controlled HIV infection on antiretroviral therapy (ART) have a fourfold increased risk of developing TB. Conversely, Mtb infection exacerbates HIV pathogenesis and increases the rate of AIDS progression. In this review, we discuss this reciprocal amplification of HIV/Mtb coinfection and how they influence each other’s pathogenesis. Elucidating the infectious cofactors that impact on pathogenesis may open doors for the design of new potential therapeutic strategies to control disease progression, especially in contexts where vaccines or the sterile clearance of pathogens are not effectively available.

## 1. Introduction

Tuberculosis (TB) is one of the oldest diseases and has been decimating human populations for millennia. In 2021 alone, there were an estimated 10.6 million active TB cases worldwide and 1.6 million deaths [1]. The total number of patients with TB includes a small proportion of latently infected people, which the WHO estimates to be a quarter of the global human population [1]. The causative bacterium, *Mycobacterium tuberculosis* (Mtb), is a facultative intracellular human pathogen that is transmitted to susceptible hosts via aerosolized secretions from the lower respiratory tract of TB patients. After an incubation period of 4 to 12 weeks, exposed individuals reached a state called latent TB infection, defined as a “persistent immune response to stimulation by Mtb antigens with no evidence of clinically manifest active TB” (https://www.who.int/publications/i/item/9789241550239, accessed on 10 February 2023). About a quarter of the world’s population is estimated to be infected with Mtb and, of these, 5–10% have a lifetime risk of progression to active TB disease [1]. The remaining 90–95% of infected individuals respond to the infection with an appropriate immune response, the hallmark of which is the formation of granulomas. Granulomas are mainly structures formed by central aggregates of infected and non-infected macrophages, lymphocytes, and neutrophils covering distinct cell layers in which Mtb is contained but not eradicated [2,3,4]. Within these structures, infected macrophages control the replication rates of the pathogen, and a few can be converted to dormant, non-replicating forms [2,5,6], leading to the establishment of a chronic latent tuberculosis infection (LTBI). Under certain circumstances, such as malnutrition or immunodeficiency, the dynamics of granuloma maintenance are disrupted, leading to uncontrolled pathogen replication and tissue destruction, culminating in tuberculosis [7]. In fact, TB is the leading cause of death in HIV-1-infected individuals [1].

Human immunodeficiency virus (HIV) type 1 and type 2 (HIV-1 and HIV-2, respectively) are the causative agents of acquired immunodeficiency syndrome (AIDS). At the end of 2021, an estimated 38.4 million people were infected with HIV (mainly HIV-1), which has claimed 40.1 million lives since the beginning of the AIDS pandemic [8].

The hallmarks of HIV infection of human hosts are: (i) the irreversible depletion of CD4+ T lymphocytes (T-CD4+ lymphocytes) both in the peripheral blood and in mucosa-associated lymphoid tissues, compromising the immunological control of microbial pathogens [9]; (ii) the establishment of a reservoir of latently infected cells (e.g., T-CD4+ lymphocytes and macrophages) carrying infectious viral genomes integrated into cell chromosomal DNA [10]; (iii) lifelong maintenance of viral replication, even in patients on antiretroviral therapy [11]; and (iv) chronic immune activation that, together with T-CD4+ lymphocyte depletion, leads to immune exhaustion, immunodeficiency, and, consequently, multiple opportunistic infections [12].

HIV-Mtb coinfection is a major public health threat, responsible for an alarming number of deaths worldwide: the WHO estimates that approximately 187,000 HIV-Mtb coinfected patients will die of tuberculosis in 2021 alone [1]. These figures reflect a synergistic effect between the deleterious outcomes of both infections, creating a new concept referred to as a synergistic epidemic or syndemic, where HIV and Mtb mutually amplify each other’s pathogenic potential. In fact, HIV-Mtb co-infected individuals are at a much higher risk of developing active tuberculosis, with estimated rates ranging from 20 to 37 times higher than the general population [13]. In addition, mortality rates are higher in HIV-infected individuals with tuberculosis compared to HIV-negative individuals with tuberculosis [14].

The mechanisms underlying the mutual exacerbation of both pathogens remain elusive, although their elucidation is critical to controlling the mortality associated with this syndemic. In this review, we address the mechanisms by which HIV infection increases the risk of active tuberculosis, focusing on the HIV infection factors that dictate the immune impairment of Mtb containment within granulomas. We also consider the Mtb-derived factors that influence HIV pathogenesis.

## 2. Main Consequences of HIV Infection

HIV is mainly transmitted through unprotected vaginal, anal, and oral sex. HIV can also be transmitted by blood transfusion, the sharing of contaminated needles, and vertical transmission from an infected and untreated mother to her child during pregnancy, childbirth, and breastfeeding. Following HIV transmission through the sexual mucosa, viral spread occurs through draining lymph nodes and the bloodstream, allowing viral infection to spread to multiple compartments of the body, namely the brain, lungs, and gut-associated lymphoid tissue (GALT) [15].

The pathogenesis of HIV infection is based on three interrelated events: (i) the ability to infect T-CD4+ lymphocytes and macrophages, which is determined by the expression of the HIV cell receptor CD4 [16,17], and a member of the chemokine receptor family acting as a co-receptor, namely, CCR5, CXCR4, or other alternative receptors [18,19]; (ii) the establishment of latently infected cells harbouring the HIV genome integrated into the chromosomal DNA of the cell [20]; and (iii) the induction of immune hyperactivation throughout infection, leading to accelerated immune senescence [21]. These three features have both local and systemic consequences, leading to lifelong infection, irreversible CD4+ T lymphocyte depletion and dysfunction, and immune senescence and exhaustion.

### 2.1. HIV as a Cytopathic Retrovirus

T-CD4+ lymphocytes and macrophages are the major target cells for HIV infection and replication in vivo. Although infection of the latter is characteristically non-cytopathic, allowing for the survival of infected macrophages with low levels of virus production throughout the cell’s life [22], infection of T-CD4+ lymphocytes invariably leads to their destruction and to an irreversible depletion of this crucial immune cell population [23]. This depletion is observed in the peripheral blood, as reflected by a decrease in circulating T-CD4+ lymphocytes [24], but it has also been documented in mucosa-associated lymphoid tissues, such as GALT [9,25].

One of the key questions in HIV pathogenesis concerns how CD4+ T lymphocytes die during HIV infection. One of the processes involved is the apoptosis of infected and bystander non-infected cells [26,27]. The death of non-infected bystander cells involves multiple mechanisms and players and includes the activation of host cell pathways (e.g., FAS ligand, TNF-α, TRAIL) that induce apoptotic events [27,28,29], and the effect of viral proteins released from infected cells that induce bystander cell death, such as Nef, Tat, Vpr, or Vpu [30,31,32,33,34].

In addition to apoptotic mechanisms, HIV contributes to CD4+ T lymphocyte depletion by the induction of pyroptosis in non-permissive CD4+ T lymphocytes [35] (discussed in more detail later in this review), and by direct cytopathic effects through the formation of syncytia, particularly in lymphoid tissues [36,37,38].

### 2.2. Establishment of Latently Infected Cells

One of the most important features of the HIV life cycle in an infected human host is resistance to eradication, even in the presence of highly active and multi-target antiretroviral drugs. In addition, when antiretroviral therapy (ART) is stopped, HIV viremia that was suppressed and undetectable during ART rebounds and returns to pre-ART levels.

This inability to cure HIV infection has been the subject of intense research and is based on HIV’s ability to infect cells that act as cellular reservoirs in multiple bodily compartments. These cells are latently infected, as defined by the absence of viral production, and consist mainly of memory CD4+ T lymphocytes, monocytes, and macrophages.

The establishment of latency is one of the strategies used by HIV to persist in infected hosts; it results from the HIV replication cycle, in which viral double-stranded DNA is retrotranscribed from genomic RNA. Under certain circumstances, this proviral DNA can be maintained in a non-transcriptional state so that HIV antigens are not expressed, and infected cells cannot be detected and targeted for a cytolytic lymphocyte response.

There are two types of latency: pre-integration latency and post-integration latency. Pre-integration latency is defined by the presence of complete or incomplete forms of viral double-stranded DNA that are not integrated into the cellular chromosomes. It appears to be quite common and occurs in resting CD4+ T lymphocytes [39,40,41,42]. However, the pre-integrated form of viral DNA in resting CD4+ T lymphocytes appears to be labile and short-lived, with a half-life of approximately one day [42], although other reports have found a longer lifespan of one week [43].

Post-integration latency in memory CD4+ T lymphocytes is considered the true latency state responsible for the lifelong persistence of HIV in an infected host. It is established after the integration step of the retroviral replication cycle and relies on the complete silencing of proviral transcription. The mechanisms underlying this non-transcriptional state include the epigenetic regulation of proviral transcription and post-transcriptional regulation (recently reviewed in [10]). Furthermore, it has been noted that the survival of latently infected CD4+ T lymphocytes in patients on long-term ART regimens depends not only on HIV gene silencing, but also on high expression levels of immune checkpoint molecules that negatively regulate T lymphocyte immune function [44,45,46,47,48].

In addition to CD4+ T lymphocytes, cells of the monocyte/macrophage lineage are also susceptible to HIV infection soon after transmission through genital and anorectal mucosa [49]. This susceptibility includes both those viruses that enter the cells through engagement of the CD4 and CCR5 chemokine receptors (R5 viruses) and those that use the CD4 and CXCR4 chemokine receptors (X4 viruses) [50,51,52]. This cell group includes peripheral blood monocytes, tissue macrophages, dendritic cells, and Langerhans cells.

Due to their functions as antigen-presenting cells and their ability to be recruited to sites of infection and inflammation, HIV-infected macrophages have been detected in several tissues and mucosa. In addition, HIV infection in macrophages is non-cytopathic, allowing for the survival of infected macrophages with low levels of virus production throughout the cell’s life [22]. Taken together, these features contribute to the establishment of a latently infected, low-level virus-producing cell population that is responsible for the creation of body sanctuaries where HIV persistence can be maintained for extended periods of time, ensuring long-term virus production even in patients on ART [53]. These HIV sanctuaries have been identified in several body compartments, including the brain, lungs, semen, urethra, liver, and several lymphoid tissues such as GALT [53].

### 2.3. Induction of Chronic Inflammation

Persistent systemic inflammation is considered one of the signatures of HIV infection and is observed even in patients on ART and with sustained suppressed viremia. Defined as the continuous (even low-level) production of pro-inflammatory cytokines and other soluble factors over long periods of time (e.g., IL-6, IL-8, which is also known as CXCL8, CCL2, which is also known as MCP-1, CCL3, CXCL10, which is also known as IP-10, IFNγ, and sCD14), chronic inflammation is responsible for severe tissue damage and an increased risk of non-AIDS comorbidities (cardiovascular disease, cancer, renal disease, neurocognitive disorders, and liver disease) and mortality [12]. These abnormally persistent and pathological levels of inflammation are multifactorial and mainly result from the direct effects of HIV-induced immune activation and from GALT-associated CD4+ lymphocyte depletion, leading to microbial translocation.

Several factors contribute to the chronic inflammation that is directly associated with HIV infection. Some originate from HIV proteins such as Nef and Vpr, which trigger immune activation [54,55]. In the case of the Nef protein, its effects may be systemic, as Nef-containing exosomes have been detected in plasma even in patients with suppressed viremia, exerting distinct effects on cells that ultimately lead to inflammation [54]. Other factors arise from the detection of viral nucleic acid by pattern recognition receptors (PRRs) during the HIV replication cycle. Both RNA and dsDNA, as well as viral proteins, are detected by PRRs [56]. HIV RNA molecules are mainly detected by retinoic acid-inducible gene I (RIG-I), while dsDNA is detected by cyclic GMP-AMP synthase (cGAS) and interferon-gamma-inducible protein 16 (IFI16), the latter playing an important role in the detection of incompletely retrotranscribed viral DNA, leading to caspase-1 activation and pyroptosis [35]. The accumulation of these incomplete viral DNA molecules is the result of an abortive replication cycle that occurs in non-activated T-CD4+ lymphocytes. Considering that 95% of the total population of T-CD4+ lymphocytes do not allow for a productive replication cycle, and are thus prone to accumulate incomplete DNA retrotranscripts, pyroptosis is not only the main pathway of cell death but also has additional consequences in the induction of potent pro-inflammatory signals [57].

Extensive damage to the intestinal mucosa is another key driver of the pathogenic chronic inflammatory response observed during HIV infection. Soon after transmission, HIV spreads to the GALT and irreversibly destroys a large proportion of mucosal-associated CD4+ T lymphocytes [9], particularly the T helper 17 (Th17) subset, which plays a critical role in promoting mucosal defence against microorganisms and barrier integrity [58]. This injury occurs during the acute phase of HIV infection, and, in contrast to the observed partial recovery of peripheral blood CD4+ T lymphocyte counts, the depletion of GALT resident cells is not reversed; this leads to mucosal dysfunction, which in turn leads to microbial translocation [59]. In fact, intestinal mucosal dysfunction allows microbes and microbial by-products from the intestinal lumen to invade the surrounding tissues and the bloodstream and also contributes to mucosal dysbiosis, which is defined as the unbalanced composition of the gut microbiota. In turn, this microbial translocation and associated dysbiosis promotes mucosal injury, further expanding and self-perpetuating HIV-induced inflammation both locally and systemically, clearly distinguishing HIV infection of human hosts from SIV infection in African non-human primates [60].

In addition, microbial translocation from the intestinal lumen into the bloodstream, as evidenced by elevated plasma levels of lipopolysaccharide (LPS), provides an additional layer of inflammatory induction [61]. LPS is a major component of the outer membrane of a significant number of Gram-negative bacteria and of some Gram-positive bacteria. LPS is sensed by Toll-like receptor 4 (TLR4), one of the PRRs present in the cell membrane, and its activation leads to intracellular signalling through NF-κB, which culminates with the production of inflammatory cytokines [62].

In conclusion, HIV infection is responsible for several direct and indirect mechanisms that lead to immune dysfunction and chronic inflammation, both local and systemic. This in turn leads to accelerated immune senescence and ageing, referred to as “inflammmageing” [63]. This scenario creates a series of deleterious factors that allow for the amplification and decontrol of several pathogens, particularly those that latently infect HIV-infected individuals, the most notable of which is *Mycobacterium tuberculosis*.

## 3. Pathogenesis of *Mycobacterium tuberculosis* Infection

**Figure 1:**
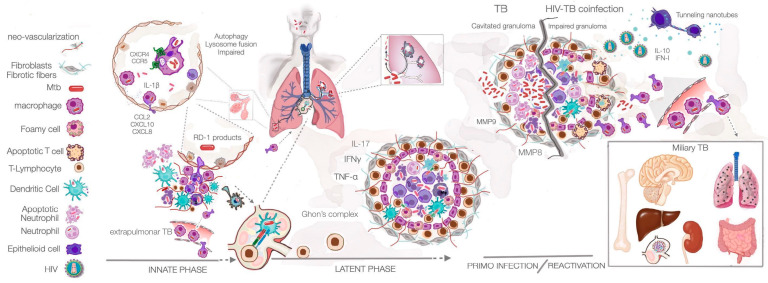
Pathogenesis of Mtb infection: implications in HIV co-infection. Inhaled aerosols containing up to three bacilli reach the alveoli in the lower part of the lung. Macrophages take up the pathogen, but Mtb impairs phagolysosome fusion and autophagy. This is enhanced during HIV co-infection, which helps Mtb to establish intracellular niches. In turn, Mtb increases the expression of the HIV coreceptors CXCR4 and CCR5 (helping HIV spread during coinfection). The secretion of IL-1β by infected macrophages and pneumocytes induces transmigration to the lung tissue. Mtb RD-1 products, together with chemokines secreted by infected macrophages, attract more innate cells, leading to the formation of the innate granuloma. If this response is strong, the infection is contained and, in 95% of people, it progresses to a latent phase. In about 5% of people, this phase leads to primary infection with pleural extrusion and eventual haematogenous dissemination. The latent granuloma is maintained by newly arrived T lymphocytes (after proper priming by dendritic cells in the mediastinal lymph nodes) and macrophages, providing a proper balance of cytokines (i.e., TNF-α, IFNγ, and IL-17). In a few granulomas, the pathogen may go into a low metabolic state of dormancy. Immunosuppressive conditions, such as malnutrition or ageing, may favour reactivation (usually in the upper part of the lung). The cytokine balance is disturbed towards increasing levels of TNF-α, leading to excessive neutrophil recruitment with the secretion of metalloproteinases (MMPs) and the caseation of the granuloma. Ultimately, after tissue cavitation, bacteria are released for transmission to new hosts. In the presence of HIV co-infection, extensive lymphocyte depletion, and low secretion of TNF-α, the granuloma dynamics and integrity are compromised. The environment of cytokines secreted by deactivated macrophages (e.g., IL-10 and IFN-I) will favour the formation of tunnelling nanotubes and the cell-to-cell spread of HIV throughout the body. We postulate that HIV-infected cells, or those under the influence of the viral Nef protein, will facilitate macrophage migration to other tissues, thereby contributing to the spread of Mtb and extrapulmonary TB.

The success of *Mycobacterium tuberculosis* (Mtb) as a pathogen depends on its ability to diversify its interactions with the immune system. It achieves this first by evading the innate response, second by surviving in the presence of a robust adaptive response (free of disease symptoms), and, finally, by inducing a strong inflammatory response that leads to extensive tissue destruction (Figure 1).

Upon reaching the alveoli, Mtb is phagocytosed by permissive, non-inflammatory alveolar macrophages, providing the appropriate intracellular environment for the establishment of latent infection [2,5,64]. The few bactericidal events of these professional phagocytic cells became even more compromised as the pathogen manipulates the fusion of the phagosome with the lysosome, the vesicle acidification, and the proteolytic activity of lysosomal cathepsins [65,66,67,68,69,70].

Pattern-associated recognition signatures of the pathogen (PAMPs), recognized by innate immune receptors (PRRs) that signal for IL-1β secretion [64,71,72], drive the translocation of infected macrophage through the alveolar pneumocyte barrier into the lung tissue, leading to the formation of an innate granuloma [4,73,74]. This is a highly dynamic structure in which controlled rates of intracellular bacterial replication induce a limited degree of host cell death [3]. A balance between the death of infected cells and replenishment by the continuous recruitment of macrophages and de novo infection from cellular apoptotic bodies expands the intracellular niches and granuloma structure [75,76]. At this stage, infected macrophages secrete cytokines (e.g., CCL2, CXCL10, TNF-α, and IL-1β) [74,77,78] and pathogen virulence factors (from the RD-1 genomic region) [2,5], which are the source of chemoattraction for more permissive macrophages from the lung interstitium or derived from peripheral blood monocytes. Other innate cells, such as neutrophils, natural killer cells (NK), and γδ T-cells, are also recruited by this gradient of chemokines. Local dendritic cells (DCs) are indeed manipulated by Mtb virulence factors from ingested free bacteria or infected cells such as macrophages or neutrophils, resulting in a delay in adaptive responses. Consequently, DC migration to the draining lymph nodes is slowed down, as is the maturation of antigen processing and presentation [79,80,81,82]. After 3–4 weeks, DCs finally reach the mediastinal lymph nodes where the priming of T lymphocytes with Mtb peptide antigens would occur in a properly effective immune response [80,83]. In addition, TB granulomas can actually acquire lymphoid functions: in the absence of secondary lymph nodes, these structures can replace this site for the priming of CD4+ and CD8+ T lymphocytes, as well as functioning as germinal centres of B lymphocytes, providing protective immunity against TB [84]. The effector cells are predominantly Th1 and Th17, as well as cytotoxic T lymphocytes [85]. All of these locally recruited immune cells form a dress coat that is involved in the biogenesis of the adaptive granuloma; together with a robust immune response, this contains the pathogen locally in the lung, and the chronic LTBI is then established (Figure 1).

The mediastinal draining lymph node and the primary lesion formed by the mature adaptive granuloma are typical hallmarks of TB, and are referred to as the Ghon’s complex [86]. During the innate phase, some infected cells disseminate from the primary lesion to seed secondary granulomas in the lung [87,88]. A typical latency granuloma is formed by a central core of infected macrophages surrounded by several layers of infected or newly formed epithelioid macrophages, foam macrophages, Langhans giant cells with an outer layer of infiltrating CD4+, and CD8+ effector or memory cells (Figure 1). Infected macrophages at the interface with infiltrating lymphocytes are licensed by this population of T lymphocytes to control intracellular bacterial growth via the secretion of TNF-α [89] and IFNγ [90,91]. TNF-α activates macrophages originating apoptosis, induces pro-inflammatory endothelial macrophage, neutrophil, and T-cell recruitment, affects macrophage phagocytosis, and activates IFNγ secretion [92]. IFNγ activates macrophages to a pro-inflammatory state, induces autophagy, oxidative bursts, and iron access restriction, and increases the expression of the MHC class II antigen presentation machinery [65,91,93,94,95,96]. IFNγ also plays a role in controlling inflammation by inducing the increased expression of indoleamine-2,3-dioxygenase (IDO) from haematopoietic and non-haematopoietic cells [97], which creates a tolerogenic state for inflammatory macrophages and dendritic cells and helps polarise T-regulatory subsets (Treg) counteracting Th17 [97]. Granulomas in a more mature stage show marked neo-vascularization and develop an extensive fibrotic capsule that delineates the border between the external lymphocytic cells and the infiltrated effector T lymphocytes [86,98,99]. Similarly, an eventual reduced ability to mount a CD4+ T-cell response is associated with the reduced maintenance of the granuloma structure and, importantly, a reduced ability to prevent metastasis of infection, similar to innate phase granuloma [76] (Figure 1). In the late stages, the centre of the granuloma loses its vascular appearance and becomes necrotic [74]. The solid adaptive granuloma is not only the site of Mtb containment during latency, but also the source of tissue damage in the early stages of the disease. An imbalance in the delicate signals of IFNγ and TNF-α contributes to extensive necrosis of cells within the granuloma and in the lung parenchyma, extensive replication of the pathogen, and the release of the bacteria into the airways, leading to transmission [100] (Figure 1).

## 4. Consequences of HIV-MTB Co-Infection in the Amplification of Pathogenesis

The detrimental effects of HIV-Mtb co-infection on the amplification of pathogenesis must be considered in two different scenarios: (i) Mtb infection in a person with pre-existing HIV infection or, conversely, (ii) HIV infection in a person with latent Mtb infection.

### 4.1. Mtb infection in a Person with Pre-Existing HIV Infection

Inadequate control of pulmonary pathogens has been a hallmark of AIDS patients since the beginning of the pandemic. The first cases of what became known as AIDS were individuals with a lung infection caused by a rare fungus, *Pneumocystis jirovecii* (formerly *Pneumocystis carinii*) [101]. Recurrent bacterial and viral pneumonia, as well as a higher risk of developing lung cancer, are consistently observed in untreated HIV-infected patients compared to uninfected individuals [102,103]. This increase in lung disease is the result of impaired local immunity due to the direct and indirect effects of chronic HIV infection, which are not repaired by ART.

As noted above, soon after transmission, HIV spreads to multiple compartments of the body, namely the lungs, brain, and GALT. In the lungs, alveolar macrophages (AMs) are the most abundant immune cells of the alveolar space and play a critical role in the control of lower respiratory tract infections. AMs express the receptors required for HIV entry (CD4, CCR5 and CXCR4) and, not surprisingly, HIV infection of these cells has been described in vivo [104,105,106,107]. Remarkably, although HIV reaches the lung compartment during the early stages of infection as a result of blood spread, analysis of lung tissue reveals the existence of viral quasispecies that are distinct from blood or lymph nodes, indicating that HIV replicates and evolves in the lung independently of other body compartments [108,109,110].

HIV infection of lung macrophages, including AMs, has a direct impact on pulmonary immunity, as it appears to impair the innate response and general phagocytic functions of alveolar and interstitial macrophages [111], including impaired autophagy [112], thus acting as a cofactor for more severe opportunistic infections [104,113]. Specifically, HIV-infected AMs show impaired phagosomal activity compared to AMs from HIV-uninfected individuals; interestingly, this impairment was observed in the entire population of AMs from the same tissue microenvironment, regardless of whether they were HIV-infected or not [104].

Furthermore, HIV infection also occurs in lung epithelial cells [114]. This compromises the integrity of the lung mucosal barrier through reduced expression of E-cadherin, which promotes paracellular permeability and triggers pro-inflammatory signals [115], which, together with the systemic pro-inflammatory mechanisms described above, contribute to additional lung abnormalities [116].

Regarding cytokine expression in lung tissue, bronchoalveolar lavage from untreated HIV-infected patients shows higher levels of RANTES and TNF-β compared to uninfected individuals, as well as a profound perturbation of the cytokine microenvironment, with a shift towards chemokine-driven networks involving SDF-1α, MIP-1α, CCL4, CCL2, CXCL10, GRO-α, eotaxin, and CXCL8. This predominance of chemoattractant cytokines in the lung during chronic untreated HIV infection is a key driver of excessive immune cell accumulation in the lung parenchyma and promotes their infiltration into the alveolar spaces, a disruptive state of local immune cell homeostasis [117].

This scenario, in which multiple factors alter normal pulmonary immunity, goes beyond the original notion that the development of TB is solely the result of HIV-induced CD4+ T lymphocyte depletion. In fact, the risk of developing TB is increased even in HIV-infected patients with controlled viremia and, more importantly, with high CD4+ T lymphocyte counts [118,119], suggesting that persistent inflammation and disrupted immune cell homeostasis are major contributors to uncontrolled Mtb infection and to TB development.

In the context of pre-existing HIV infection, it is important to emphasize that early control of Mtb infection relies on phago-lysosomal activity by AMs and other proper innate immune responses, including autophagy (Figure 1); these are conditions that, as noted above, are impaired by virus-induced factors. In addition, HIV infection disrupts several important mechanisms involved in the initial steps of early Mtb control. For example, natural killer (NK) cells from HIV-infected individuals have reduced production of IFNγ, IL-15, and granzyme B in response to Mtb infection [120], and IFNγ production is impaired in peripheral blood mononuclear cells from HIV-infected individuals [121]. Furthermore, the viral envelope glycoprotein gp120 is a potent inducer of IL-4 and IL-13 [122], two cytokines responsible for macrophage polarisation towards an anti-inflammatory M2 phenotype that promotes Mtb survival and replication by interfering with the mitochondrial metabolism [123]; it also promotes metaplasia to an epithelioid macrophage phenotype that contributes to the expansion of innate granulomas [3,76].

Thus, the aforementioned mechanisms related to the impaired phagosomal activity of HIV-infected AMs, together with the reduced innate response and impaired antigen presentation and T-cell activation [104,113], affect the host’s ability to control Mtb infection, resulting in dysfunctional innate granulomas with increased bacterial replication and metastatic dissemination to foreign organs and tissues [124].

TB is known to facilitate HIV replication from viral sanctuary niches, contributing to the progression of HIV to AIDS [125]. The contribution of LTBI to HIV progression is indeed supported by Mtb-induced immune activation and changes in the inflammatory milieu at infected sites with systemic effects [126]. In fact, it is reported that, during Mtb infection, immune responses contribute to the increased replication of HIV-1 in the blood [127] and at sites of bacterial infection in the lungs [128]. Increased HIV replication has also been observed in activated cells such as lymphocytes and macrophages in the pleural space [129,130]. Regarding alveolar and interstitial macrophages, or blood-derived arriving macrophages, Mtb induces the expression of both coreceptors for HIV CCR5 and CXCR4, promoting viral infection in these host cells and the consequent expansion of viral reservoirs [131] (Figure 1). Mtb virulence factors, such as the wall glycolipid LAM, have been shown to induce the secretion of proinflammatory cytokines including TNF-α [131], which activate transcription factors such as AP-1 and NF-κB in CD4+ T lymphocytes and macrophages harbouring proviral DNA, resulting in the transcriptional activation of HIV long terminal repeats (LTRs) and the production of new viral particles [132,133,134]. The main consequences of HIV infection for the function of various cells and tissues are summarized in Table 1.

### 4.2. HIV Infection in a Person with Latent Mtb Infection

The pathogenesis of Mtb infection described above clearly shows that the formation and maintenance of granulomas is an essential feature that distinguishes latent Mtb infection from TB (Figure 1). It is also clear that the cell composition and cytokine balance are critical not only for granuloma formation but also for the maintenance of granuloma integrity. This dependence on the granuloma structure to keep Mtb under control is an obvious Achilles’ heel for the host. Any event that disrupts this delicate balance of pro/anti-inflammatory signals could potentially lead to increased Mtb replication, the disruption of the granuloma structure, and the release of new bacilli capable of infecting new cells, which spread to different compartments (Figure 1) and are eventually transmitted to new hosts in aerosolized respiratory secretions.

In a pre-existing Mtb infection, specifically within the granuloma, HIV encounters a highly permissive CD4+ T lymphocyte population expressing the membrane receptors required for viral infection (CD4, CCR5, or CXCR4). As a result of infection, these CD4+ T lymphocytes (e.g., Mtb-specific T lymphocytes) are destroyed by apoptosis, contributing to the breakdown of granuloma integrity, a decrease in Mtb control, and an increase in the bacterial load [7,135,136]. In addition, HIV infection alters the balance between anti-inflammatory and pro-inflammatory signals in the macrophage population, with a change in the cytokine composition milieu within the granuloma; this feature increases lung inflammation and, more importantly, contributes to granuloma disintegration [115,117,137,138]. Finally, the immune response to Mtb induces the recruitment of additional fully HIV-permissive CD4+ T lymphocytes to the granuloma, namely CCR5+ CD4+ T lymphocytes and macrophages [131,139,140,141], thereby increasing virus production, which in turn infects and destroys more cells. This vicious, self-perpetuating cycle helps to compromise Mtb containment within the granuloma and facilitates the dissemination of the pathogen to other tissues. In particular, apoptosis of the Th17 subset, which is required for IL-17 secretion and neutrophil recruitment, is implicated in the impaired development of necrotic granulomas [142] (Figure 1). The remodelling events leading to lung cavitation are largely controlled by the neutrophil-derived metalloproteinase MMP-8 [143]. In fact, HIV-infected individuals tend to develop smaller and fewer necrotic granulomas than immunocompetent individuals; although they are more susceptible to TB and have miliary forms of the disease (Figure 1), they tend to transmit fewer bacilli due to the lower proportion of smear-positive TB cases [144,145].

In addition, the HIV Nef protein has been shown to modify the migratory capacity of infected cells, facilitating viral dissemination to different organs and tissues of the human body by enhancing macrophage mesenchymal migration [146]. We postulate that this may also facilitate the tissue metastasis of Mtb-infected macrophages during co-infection (Figure 1). In some respects, HIV-1 causes a disease that more closely resembles primary progression in infants [147].

During LTBI, the increased expression of indolamine-2,3-dioxygenase (IDO) by macrophages and dendritic cells leads to an anergic state in T lymphocytes [148,149], with a concomitant decreased secretion of IFNγ, which promotes viral and bacterial replication [150]. Furthermore, as a consequence of any chronic infection, T lymphocyte exhaustion promotes irresponsiveness to infected cells, preventing their eradication [151]. In addition, chronic infections such as those caused by Mtb or HIV result in the prolonged secretion of type I interferon (IFN-I) [152,153,154]. IFN-I induces several relevant antimicrobial responses during the innate immune phase of infection, either against viruses or intracellular bacteria [153,155,156,157,158]. However, during chronic HIV infection, continuous IFN-I production impedes immune recovery and enhances systemic immune dysregulation [159]. For Mtb, while the effects of IFN-I are harmless during the innate and latent controlled phase, they have been shown to be hostile pathogenic, with a deleterious role in promoting disease progression. In TB patients, blood transcriptional gene signatures for type I IFN-related genes have been found to correlate with disease severity [160]. In animal models, they are associated with extensive recruitment of neutrophils into the lungs, which is associated with damaging tissue pathology [161]. In addition, type I IFNs are a driver of IL-10, a cytokine that impairs antimycobacterial immune responses [162].

Consequently, chronic IFN-I secretion with a concomitant increase in IL-10 induces the polarisation of pro-inflammatory macrophages and dendritic cells into an immune-deactivated state. These, in turn, have a greater effect on the secretion of immunosuppressive cytokines such as IL-10 and TGFβ [153,163]. A secondary effect of the excessive secretion of both cytokines is the fibrosis often seen in lymph nodes, which impairs their function and contributes to immunodeficiency and AIDS progression. [149]. Moreover, a microenvironment enriched in IL-10 and IFN-I (such as the latent adaptive Mtb granuloma) provides an ideal permissive environment for HIV replication. Indeed, Mtb has been shown to exacerbate HIV infection by promoting direct cell-to-cell viral transfer through the formation of tunnelling nanotubes induced in macrophages stimulated by both cytokines [164,165] (Figure 1). The main effects of Mtb infection on the function of different cells and tissues are summarized in Table 2.

**Table 1:** HIV-induced changes in various cells and tissues that directly affect the immune response to Mtb.

Cell or Tissue	Changes Induced by HIV Infection	References
CD4+ T lymphocytes	Apoptosis, which induces the death of infected and bystander cells; pyroptosis	[26,27,35]
Alveolar macrophages	Impaired phagosomal activity	[111,112]
Lung epithelial cells	Reduced expression of E-cadherin, which promotes paracellular permeability and triggers pro-inflammatory signals	[115]
Lung tissue	Shift in the cytokine microenvironment towards chemokine-driven networks involving SDF-1α, MIP-1α, MIP-1β, CCL2, CXCL10, GRO-α, eotaxin, and CXCL8	[117]
NK	Reduced production of IFNγ, IL-15, and granzyme B in response to Mtb infection	[121]
Macrophages	The viral protein gp120 induces the production of IL-4 and IL-13, which are responsible for polarisation towards an anti-inflammatory M2 phenotype	[122,123]
Neutrophils	Death of Th17 CD4+ T lymphocytes impairs the recruitment of neutrophils and the development of necrotic granulomas	[142]
Alveolar macrophages	Nef viral protein enhances macrophage mesenchymal migration, facilitating viral spread to multiple organs	[146]

## 5. Conclusions

Co-infections are a source of increased morbidity and mortality, and the outcomes of co-infections are generally worse than those of single-pathogen infections. This occurs either because the pathogens involved target the same organ or tissue, resulting in cell death and tissue damage that can potentiate the effects of the other pathogen, or because they target similar cellular functions, mutually amplifying their effects, or because they disrupt mechanisms that are important for reciprocal control.

HIV/Mtb co-infection is a clear example of the damaging synergy between a virus that induces immunodeficiency and a bacterium whose control depends heavily on the host’s ability to limit its replication and spread. The human host and the Mtb have co-evolved to ensure that both survive without excessive side effects. However, this delicate balance is easily disrupted when immune control is compromised (Figure 1).

HIV is a master at suppressing the host immune response (Table 1). Even with highly effective ART, HIV infection is untreatable and viral replication is not suppressed. By directly inducing CD4+ T lymphocyte depletion or by establishing a chronic inflammatory environment, HIV is the main driver of Mtb proliferation and the conversion of latent Mtb infection to tuberculosis. Furthermore, the granuloma within which Mtb is maintained and controlled is a source of fully permissive cells that HIV can use to efficiently replicate and destroy, or else latently infect and use as a ‘Trojan horse’ to invade other body compartments. This in turn promotes further immune activation and inflammation, creating a self-sustaining mechanism that triggers Mtb replication and decontrol that is difficult to stop or even reduce.

Although it lies outside the scope of this review, it is also important to mention the paradoxical effect observed in HIV/Mtb co-infected patients after the initiation of ART, referred to as immune reconstitution inflammatory syndrome (IRIS). This syndrome results in an exacerbation of the patient’s immune response to various pathogens, but particularly to Mtb, and is the result of a rapid restoration of immune competence observed as a consequence of ART (reviewed in [166]).

In addition to these events, the increased replication of both pathogens favours the emergence of new variants with different phenotypic characteristics, in particular, the selection of drug-resistant strains that eliminate the ability to control their replication and to treat the disease, such as the XDR variants of Mtb.

Understanding the mechanisms underlying the pathogenesis of HIV and Mtb during co-infection is crucial for preventing the synergistic effects of both pathogens and for identifying better targets for the development of more effective drugs to treat these infections.

## Figures and Tables

**Table 2:** Mtb-induced changes in various cells and tissues that directly affect the immune response and control of HIV infection.

Cell or Tissue	Changes Induced by *Mycobacterium tuberculosis* (Mtb) Infection	References
Peripheral blood	Immune responses to Mtb contribute to increased HIV replication through the activation of macrophages and CD4+ T lymphocytes	[127]
Lungs	Immune responses to Mtb contribute to increased HIV replication through the activation of macrophages and CD4+ T lymphocytes	[128]
Granuloma	Recruitment of CD4+ T lymphocyte and macrophage populations expressing membrane receptors required for viral infection	[131,139,140,141]
Alveolar and interstitial macrophages	Induces the expression of HIV coreceptors CCR5 and CXCR4, promoting viral infection of these cells	[131]
Macrophages and CD4+ T lymphocytes	The Mtb wall glycolipid LAM induces the secretion of pro-inflammatory cytokines that activate transcription factors in CD4+ T lymphocytes and macrophages harbouring proviral DNA, leading to the transcriptional activation of integrated proviral DNA and the production of new viral particles	[131,132,133,134]
Macrophages and dendritic cells	Increased expression of IDO, leading to an anergic state in T lymphocytes, with the concomitant decreased secretion of IFNγ, which promotes viral replication	[148,149,150]
Several compartments	Prolonged secretion of IFN-I, which induces the polarisation of pro-inflammatory macrophages and dendritic cells into an immune-deactivated state via immunosuppressive cytokines such as IL-10 and TGFβ. Both cytokines are associated with fibrosis in lymph nodes, impairing their function and contributing to immunodeficiency and AIDS progression	[152,153,154,163]
Macrophages	Promotes direct cell-to-cell viral transfer through the formation of tunnelling nanotubes induced in macrophages stimulated by IL-10 and IFN-I	[164,165]
